# Effectiveness of a brief behavioral intervention for insomnia (BBII) during the COVID-19 pandemic: Mexican case report

**DOI:** 10.5935/1984-0063.20200055

**Published:** 2020

**Authors:** Horacio Balam Álvarez-García, Ulises Jiménez-Correa, Katie Moraes de Almondes

**Affiliations:** 1 National Autonomous University of Mexico, Sleep Disorders Clinic, Medicine Faculty, Research Division - Mexico City - Mexico.; 2 National Autonomous University of Mexico, Postgraduate program in Behavioral Neuroscience, Psychology Faculty - Mexico City.; 3 Onofre Lopes University Hospital, AMBSONO Sleep Clinic - Natal - Rio Grande do Norte - Brazil.; 4 Federal University of Rio Grande do Norte, Department of Psicobiología and Postgraduate Program in Psicobiología - Natal - Rio Grande do Norte - Brazil.

**Keywords:** Sleep Initiation and Maintenance Disorders, Cognitive Behavioral Therapy, Pandemics, Coronavirus, Psychotherapy, Brief, Case Reports

## Abstract

This report describes a case of a 42-year-old man. Due to overwork, initially has developed insufficient sleep syndrome; and later insomnia that temporarily coincided with the COVID-19 pandemic. A brief behavioral intervention for insomnia (BBII) was implemented that included: sleep restriction therapy (SRT), stimulus control therapy (SCT), sleep hygiene, and progressive muscle relaxation (PMR). The intervention was designed as five weekly sessions; nevertheless, it should be mentioned that starting with the third consultation, telepsychology was started due to the recommendations for social isolation implemented by the COVID-19 pandemic. At the end of treatment, the patient increased time and subjective sleep quality. Despite the social distancing measures (which started in the middle of the treatment), the patient had recovery of the sleep quality, highlighting the importance of implementing the telepsychology during the COVID-19 quarantine.

## INTRODUCTION

Insomnia disorders are characterized by the complaint of persistent difficulty with sleep initiation, duration, consolidation, or quality that occurs despite adequate opportunity and circumstances for sleep. These symptoms should cause clinically significant impairment in social, occupational, educational, academic, and behavioral functioning^1^.

Also, insomnia has been explained through the interaction between predisposition, precipitation and perpetuation factors^2^. Chronic insomnia and associated daytime symptoms occur at least several 3 times per week for at least 3 months, with sleep onset latencies and periods of wakefulness during sleep >30 minutes in middle and older aged adults^1^.

Insomnia has become a common disorder that is affecting a large global population and impairing the general health and mental wellbeing; mainly during home confinement due to pandemic COVID-19^3^. Estimates of insomnia prevalence vary between countries, but about 35% of the world’s population suffers from insomnia^4^. Insomnia prevalence of 35.0% has been reported in Mexico City^5^.

It has been argued that psychological treatment should be used as the initial intervention, due to its high degree of effectiveness, easy application, low cost, no risk of side effects or addiction to psychotropic drugs^6^^-^^9^.

This intervention has been called cognitive behavioral therapy for insomnia (CBT-I), composed for techniques such as stimulus control therapy (SCT), sleep restriction therapy (SRT), relaxation techniques, cognitive restructuring and sleep hygiene SH practices^6^. The standard treatment consists of 12 weekly sessions, having a high level of therapeutic efficiency^10^.

Over time, short versions have been proposed. For example, the brief behavioral therapy for insomnia (BBTI) is a protocol that emphasizes SCT, SRT, progressive muscle relaxation PMR and SH practices^11^. Consisting of 4 sessions, two face-to-face and two telephone sessions, this protocol has shown effectiveness similar to the standard treatment^12^. To date, the sleep disorder clinic of the national autonomous university of Mexico, is one of the few sleep centers that offers CBT as the first option treatment for insomnia in Mexico. In this direction, the aim was to describe a clinical case report in which was carried out a brief online behavioral intervention for insomnia during pandemic COVID-19. Our hypothesis is that the telepsychology will benefit patients with insomnia and could be effective in controlling symptoms.

## CASE REPORT

We present the case of a 42-year-old male patient (D.), single and living alone. He currently works as a lawyer in a law firm and is a professor at the undergraduate level.

The reason for consultation was insomnia lasting 4 months, characterized by sleep-onset and sleep-maintenance insomnia (subjective sleep latency of up to 3 hours, and 3 or 4 awakenings of 30 minutes on average, respectively). He also complained of daytime sleepiness and tiredness; therefore, began consuming energy drinks.

In addition to insomnia, at the initial consultation, he denied symptoms of other sleep disorders or any other diagnosed illness. There are no previous or current treatments for insomnia.

D. mentions that since 6 months ago initiated with greater workload; and in addition to his activities in the office, he began to perform work at home. The result was a later bedtime (3:00 a.m.) which led to chronic sleep restriction.

After two months, the workload decreased but D. maintained the consumption of energy drinks during the afternoon, staying in the bedroom using personal computer and cell phone lying down, also going to bed regularly between 2:00 a.m. and 3:00 a.m., when D. started to go to bed at 11:00 p.m. began insomnia symptoms. This situation has been maintained for the last 4 months.

## MATERIAL AND METHODS

**Participant:** The patient provided written informed consent allowing for the use of his clinical data in the report.

**Design:** The screening included a structured interview. Sleep psychologist applied to the patient a sleep diary (weekly) to identify the baseline and changes in sleep quality and subjective sleep time, sleep quality index Pittsburgh (PSQI)^13^; Athens insomnia scale (AIS)^14^. An intervention was planned in order to control nocturnal and diurnal insomnia symptoms. It was integrated by 5 weekly sessions structured according to the Table 1. A month after the fifth session, a follow-up telephone intervention was given in which the PSQI and AIS were applied again.

**Table 1:** Description and weekly planning of the sessions of the BBII.

	Week 1	Week 2	Week 3	Week 4	Week 5
1st Session:					
	Evaluation with a structured interview and application of the validated versions of PSQI 13, AIS 14 and a Sleep Diary; and initiated with SH practices.				
2nd Session		Initiation of SCT and SRT.			
3rd Session			Start of the PMR.		
4th session				Telephone follow-up.	
5th session					Telephone monitoring and application of the PSQI and AIS.

BBII Brief Behavioral Intervention for Insomnia; PSQI Pittsburgh Sleep Quality Index; AIS Athens Insomnia Scale; SH Sleep Hygiene; SCT Stimulus Control Therapy; SRT Sleep Restriction Therapy; PMR Progressive Muscular Relaxation^13^^,^^14^.

## RESULTS

As can be seen in Table 2, through the BBII sessions, there were an increase in the subjective sleep efficiency along with a decrease in the number of insomnia nights per week, in the subjective sleep latency and the number of awakenings per night. The patient also have a decrease in diurnal symptoms of tiredness and sleepiness per week. In addition, improvement in insomnia symptoms can be observed with decreases in the total scores obtained of AIS and PSQI.

**Table 2:** Subjective indicators of sleep quality and total score of the scales applied through the BBII sessions.

	Session No 1	Session No 2	Session No 3	Session No 4	Session No 5	Telephone follow-up
BT	23:00 a.m.	12:00 a.m.	12:00 a.m.	12:00 a.m.	12:00 a.m.	12:00 a.m.
WT	6:30 a.m.	6:30 a.m.	6:40 a.m.	6:30 a.m.	7:00 a.m.	7:00 a.m.
SSE %	56.0	84.6	90.0	95.3	87.1	87.1
NI / 7	5	3	2	1	1	0
SSL min	45	30	15	15	10	10
NA / night	3	1	1	0	0	0
DT / 7	5	2	3	0	0	0
DS / 7	5	2	3	0	0	0
AIS	15	-	-	-	3	3
PSQI	14	-	-	-	5	5

**BT** Bed Time, **WT** Wake up Time, **SSE** % Subjective Sleep Efficiency, **NI** /7 Nights of Insomnia per week, **SSL min** Subjective sleep latency in minutes, **NA** Number of awakenings per night, **DT** / 7 Daytime Tiredness symptom per week, **DS** / 7 Daytime sleepiness symptom per week, **AIS** Athens Insomnia Scale, **PSQI** Pittsburgh Sleep Quality Index.

**Figure 1:**
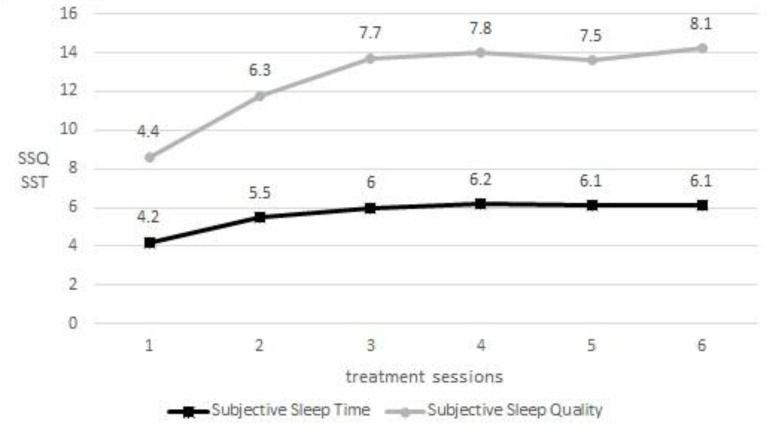
Weekly average of subjective sleep quality (SSQ) and subjective sleep time (SST). The gray line represents the subjective sleep quality (#/10) and the black line represents the subjective sleep time (hours) during BBII.

Furthermore, in Figure 1 can be observed the progressive increase in time and sleep quality evidencing the improvement of the patient through the intervention.

## DISCUSSION

It is very important to consider that the non-pharmacological treatment of insomnia is a much safer option than pharmacologic treatment. It allows avoid the abuse in the consumption of drugs, as well as loss of therapeutic efficiency and withdrawal symptoms^15^.

Consistent with the literature^16^^,^^17^, as progressed the weeks of treatment a gradual increase in sleep efficiency was observed in accordance with the decrease on night and daytime insomnia symptoms. This could be in response to the gradual inclusion of the SH practices, SCT, SRT and PMR in each of sessions.

Very important is the fact that this is a case in which there was good recovery, despite the onset of confinement caused by the quarantine by COVID-19, and the need to continue treatment using the telepsychology modality from the 3^(rd)^ consultation.

Brief behavioral intervention for insomnia through of the telepsychology should be considered as a practical psychotherapeutic option during the pandemic. Our study shows that patient had an improvement in time and sleep quality. This strategy is likely to become a mandatory working procedure for sleep disorder clinics for patients with insomnia during the COVID-19 pandemic.

## References

[1] American Academy Sleep Medicine (AASM) (2014). International classification of sleep disorders (ICSD-3).

[2] Spielman AJ, Caruso LS, Glovinsky PB (1987). A behavioral perspective on insomnia treatment. Psychiatr Clin North Am.

[3] Altena E, Baglioni C, Espie CA, Ellis J, Gavriloff D, Holzinger B (2020). Dealing with sleep problems during home confinement due to COVID-19 outbreak: practical recommendations from a task force of the European CBT-I Academy. J Sleep Res.

[4] Morin CM, Jarrin DC (2013). Epidemiology of insomnia. Sleep Med Clin.

[5] Bouscoulet LT, Vazquez-Garcia JC, Muiño A, Marquez M, López MV, Oca MM (2008). Prevalence of sleep related symptoms in four Latin American cities. J Clin Sleep Med.

[6] Baglioni C, Altena E, Bjorvatn B, Blom K (2020). The European academy for cognitive behavioural therapy for insomnia: an initiative of the European insomnia network to promote implementation and dissemination of treatment. J Sleep Res.

[7] Morin C (2006). Cognitive-behavioral therapy of insomnia. Sleep Med Clin.

[8] Riemann D, Baglioni C, Basetti C, Bjorvatn B, Groselj LD, Ellis J (2017). European guideline for the diagnosis and treatment of insomnia. J Sleep Res.

[9] Schutte-Rodin S, Broch L, Buysse D, Dorsey C, Sateia M (2008). Clinical guideline for the evaluation and management of chronic insomnia in adults. J Clin Sleep Med.

[10] Cheung JMY, Jarrin DC, Ballot O, Bharwani AA, Morin CM (2019). A systematic review of cognitive behavioral therapy for insomnia implemented in primary care and community settings. Sleep Med Rev.

[11] Germain A, Buysse D, Perlis M, Aloia M, Kuhn B (2011). Brief behavioral treatment of insomnia. Behavioral treatments for sleep disorders. A comprehensive primer of behavioral sleep medicine interventions.

[12] McCrae CS, Bramoweth AD, Williams J, Roth A, Mosti C (2014). Impact of brief cognitive behavioral treatment for insomnia on health care utilization and costs. J Clin Sleep Med.

[13] Jiménez-Genchi A, Monteverde-Maldonado E, Nenclares-Portocarrero A, Esquivel-Adame G, Vega-Pacheco A (2008). Confiabilidad y análisis factorial de la versión en español del índice de calidad de sueño de Pittsburgh en pacientes psiquiátricos. Gac Méd Méx.

[14] Nenclares-Portocarrero A, Jimenez-Genchi A (2005). Estudio de validación de la traducción al español de la escala atenas de insomnio. Salud Mental.

[15] Pottie K, Thompson W, Simon D, Grenier J, Sadowski CA, Welch V (2018). Deprescribing benzodiazepine receptor agonists: evidence-based clinical practice guideline. Can Fam Physician.

[16] Troxel W, Germain A, Buysee D (2012). Clinical management of insomnia with brief behavioral treatment (BBTI). Behav Sleep Med.

[17] Gunn H, Tutek J, Buysse D (2019). Brief behavioral treatment of insomnia. Sleep Med Clin.

